# Editorial: Effects of nonpharmacological interventions on HbA1c variability and glycemic control

**DOI:** 10.3389/fendo.2024.1482920

**Published:** 2024-10-21

**Authors:** João Soares Felício

**Affiliations:** Endocrinology Division, University Hospital João de Barros Barreto, Federal University of Pará, Belém, Brazil

**Keywords:** glycemic control, HbA1c, diabetes mellitus, nonpharmacoligical management, nonpharmacological interventions

Multiple factors impact glycemic management, but collaboratively developed treatment plans and a healthy lifestyle can significantly improve disease outcomes and well-being. Lifestyle management and psychosocial care are the cornerstones of diabetes management, and building positive health behaviors and maintaining psychological well-being are foundational for achieving diabetes treatment goals and maximizing quality of life (1). Essential to achieving these goals are diabetes self-management education and support (DSMES), nutrition therapy, routine physical activity, health behavior counseling, and psychosocial care (2).

Among the main types approaches for diabetes mellitus (DM), the lifestyle change based on nutritional interventions and physical exercise stands out. Multiple kinds of physical activity have been shown to enhance health and glycemic management in people with Type 2 DM (T2DM) (3). The role of macronutrients and other metabolites in the development of T2DM have been studied extensively (4) and there are many dietary patterns available for T2DM patients, including low carbohydrate diets, mediterranean diet, vegan/vegetarian diets, intermittent fasting and macrobiotic diets (5–7). Furthermore, dietary minerals, trace elements and vitamins can alter blood glucose and cellular glucose metabolism, and several micronutrients are associated with the risk and progression of T2DM, reinforcing the importance of studies testing the impact of dietary micronutrients in DM (8). This Research Topic groups five articles regarding the effects of nonpharmacological interventions on HbA1c variability and glycemic control.

Ren et al. conducted a systematic review and meta-analysis to investigate the impact of fruit consumption on glycemic control. The authors conducted a literature search across several databases aiming to identify randomized controlled trials that investigated the effects of fruit consumption on glucose control. The authors included a total of 19 randomized controlled trials, involving 888 participants, and found that fruit consumption led to a significant decrease in fasting blood glucose concentration, but no significant difference in HbA1c levels. The authors suggested that including more fruits in the diet could be beneficial for individuals with diabetes.

Uliana et al. conducted a cross-sectional study to investigate the relationship between Carbohydrate Counting (CC) practice and glycemic control in adults with Type 1 Diabetes Mellitus (T1DM) in Brazil. An online form was used to collect data and a logistic regression analysis were applied. Participants who were undergoing CC practice were 3 times more likely to have adequate HbA1c. The authors highlighted the benefit of this strategy.

Su et al. conducted a systematic review and meta-analysis to investigate the effects of resistance training (RT) on HbA1c and Fast Blood Glucose (FBG) levels in patients with T2DM. The authors pointed out the lack of specific dose-response relationships in the literature and aimed to determine the overall effects of RT on HbA1c and FBG and to establish dose-response relationships for RT variables, analyzing randomized controlled trials (RCTs). The authors analyzed data from 26 studies including a total of 1336 participants. They found that the most pronounced reductions in HbA1c and FBG occurred with specific training parameters, such as a training duration of 12-16 weeks, intensities of 70-80% of 1 RM (one-repetition maximum), training frequencies of 2-3 times per week, 3 sets per session, 8-10 repetitions per set, and less than a 60-second rest interval. The article concludes that RT has a beneficial impact on HbA1c and FBG levels in T2DM patients. Furthermore, the critical training parameters identified in the study are deemed essential for optimizing HbA1c and FBG reductions, providing guidance for clinical staff in designing RT exercise regimens for T2DM patients. Additionally, the findings of Ribeiro et al. conducting a systematic review to analyze the relationship between physical training variables and glycemic control in T2DM, reinforced the exercise benefit, including aerobic, resistance, and combined training interventions.

Rådholm et al. conducted a study to evaluate the potential impact of dog ownership on glycemic control, achievement of treatment goals, and all-cause mortality in individuals diagnosed with type 2 diabetes. The authors concluded that owning a dog at the time of diagnosis with type 2 diabetes did not lead to improved achievement of treatment targets or reduced mortality risk. They found that dog ownership was associated with a smaller reduction in HbA1c and a reduced likelihood of reaching treatment goals for HbA1c, LDL, and SBP.

Finally, Azulay et al. guided a cross-sectional study with 152 participants to evaluate the influence of adherence to diet and regular exercise on achieving good glycemic control in patients with T1DM and whether the percentage of genomic ancestry exerts influence on this. These findings reinforce that consistent adherence to dietary regimens is associated with good glycemic control, even regarding the adjustment for sociodemographic and genomic ancestry factors in an admixed population of T1DM patients from Northeast Brazil.

Thus, even with the development of increasingly efficient drugs for the control of DM, including Retatrutide, an agonist of the glucose-dependent insulinotropic polypeptide, glucagon-like peptide 1 and glucagon receptors, which was shown in a recent phase 2 clinical trial to be able to reduce up to 24.2% of the weight of research participants (9). It is noted that nonpharmacological interventions are still one of the cornerstones of diabetes management and must be allied to pharmacological approaches. Therefore, researches involving nonpharmacological interventions should be valued in order to make the management of patients with DM more integrative and efficient.
